# VISTA-mediated immune evasion in cancer

**DOI:** 10.1038/s12276-024-01336-6

**Published:** 2024-11-01

**Authors:** Raymond J. Zhang, Tae Kon Kim

**Affiliations:** 1 Medical Scientist Training Program, Vanderbilt University School of Medicine, Nashville, TN USA; 2 Program in Cancer Biology, Vanderbilt University, Nashville, TN USA; 3 Division of Hematology/Oncology, Department of Medicine, Vanderbilt University Medical Center, Nashville, TN USA; 4 Vanderbilt Center for Immunobiology, Vanderbilt University Medical Center, Nashville, TN USA; 5 Department of Pathology, Microbiology, and Immunology, Vanderbilt University Medical Center, Nashville, TN USA; 6 Vanderbilt Ingram Cancer Center, Nashville, TN 37232 USA

**Keywords:** Immune evasion, Translational research

## Abstract

Over the past decade, V-domain immunoglobulin suppressor of T-cell activation (VISTA) has been established as a negative immune checkpoint molecule. Since the role of VISTA in inhibiting T-cell activation was described, studies have demonstrated other diverse regulatory functions in multiple immune cell populations. Furthermore, its relevance has been identified in human cancers. The role of VISTA in cancer immune evasion has been determined, but its mechanisms in the tumor microenvironment remain to be further elucidated. Understanding its contributions to cancer initiation, progression, and resistance to current treatments will be critical to its utility as a target for novel immunotherapies. Here, we summarize the current understanding of VISTA biology in cancer.

## VISTA’s role in cancer immune evasion explored

Cancer immune surveillance, the body’s method of using the immune system to find and kill cancer cells, is sometimes thwarted, leading to tumor growth. This review focuses on a protein named V-domain immunoglobulin suppressor of T-cell activation (VISTA) and examines how it may help cancer cells avoid immune detection. Studies with both human and mouse cells have identified how VISTA works and its effect on immune cells. Mouse model experiments and analyses of human cancer samples have also investigated how VISTA affects the immune system’s cancer-fighting ability. Together, these studies suggest that high VISTA levels in immune cells can hinder the immune response against tumors, aiding cancer cell growth and spread. Therefore, future strategies targeting VISTA could improve the immune system’s ability to find and kill cancer cells and require further investigation. This summary was initially drafted using artificial intelligence, then revised and fact-checked by the author.

## Introduction

Cancer immune surveillance is the process by which the immune system identifies and eradicates cancerous and precancerous cells. However, tumors can still develop through the process of immune evasion, where cancerous cells can employ numerous mechanisms to avoid or suppress attacks from the immune system. Recent strategies in cancer treatment highlight the importance of targeting these immune-evasive mechanisms, which in turn activates the immune system to eradicate cancer cells. Namely, immune checkpoint inhibitors targeting the cytotoxic T lymphocyte-associated protein 4 (CTLA-4) and programmed cell death-1 (PD-1) pathways have led to significant improvements in patient outcomes. However, many cancers remain unresponsive to these emerging therapies, and immune-related adverse events are observed in many patients receiving these checkpoint inhibitors^1^. These shortcomings reveal the limitations of the currently available immunotherapies as well as our incomplete understanding of the immune mechanisms at play in cancer. In response, there has been a growing effort to elucidate these unknown mechanisms of immune resistance to identify novel targets of cancer immunotherapy.

V-domain immunoglobulin suppressor of T-cell activation (VISTA, programmed death-1 homolog [PD-1H], DD1α, Gi24, Dies-1) is a type I transmembrane protein that acts as an immune-regulatory checkpoint on hematopoietic cells and shares homology with both the B7 family ligand programmed death-ligand 1 (PD-L1) and the CD28 family receptor PD-1. Like other members of the B7 family of ligands, VISTA contains a conserved immunoglobulin variable region (IgV)-like domain, and this IgV-like domain of VISTA shows the highest homology with that of PD-L1 (22% sequence identity)^2^. However, the overall structure of VISTA, which contains only a single IgV-like domain, more closely resembles the single IgV structure of CD28 family receptors. In fact, a phylogenetic analysis of full-length VISTA revealed its homology to PD-1^3^. Structurally, VISTA is more highly conserved between mice and humans than all other members of the B7 family are, with 76% sequence identity and up to 91% identity of the cytoplasmic domain^4^. This homology suggests conserved functions, which is corroborated by studies demonstrating largely comparable roles for both human and mouse VISTA^5–7^.

Among all subsets of hematopoietic cells, myeloid cells, particularly monocytes, granulocytes, and dendritic cells (DCs), display the highest expression of VISTA. In lymphocytes, there is modest VISTA expression by T cells but no VISTA expression by B cells^2,3,6^. In contrast to most other immune checkpoints, which are upregulated during different stages of stimulation or activation, VISTA is expressed by immune cells at steady state. This constitutive expression across a wide range of immune cells is unique and suggests that VISTA plays a role in maintaining immune system homeostasis.

Studies investigating the role of VISTA expression in cancer have supported its function as an immunosuppressive checkpoint that impairs antitumor immunity^5,7^. However, the proposed mechanisms by which this immune suppression is affected vary. These mechanisms may be further complicated by the fact that VISTA can function as both a receptor and a ligand and that it may have distinct actions across different cell types. Ultimately, it is most likely the sum of these mechanisms, including those yet to be described, that determine the overall role of VISTA. Thus, a better understanding of the distinct mechanisms of VISTA will help clarify its exact molecular functions in the context of cancer and may facilitate its development as a promising target for immunotherapy. This review summarizes the roles of VISTA in T cells and myeloid immune cells and discusses how these currently known mechanisms may impact cancer immunity and progression.

## Negative checkpoint regulation of T cells in cancer

VISTA is perhaps most well characterized in relation to T cells. When expressed either as a receptor on the surface of T cells or as a ligand on the surface of antigen-presenting cells (APCs) or target cells, VISTA can contribute to immune tolerance by suppressing T-cell activity^2,8^. Evidence also suggests that VISTA expression significantly contributes to tumor immune evasion in the tumor microenvironment (TME) via these T-cell-suppressive functions. Although several mechanisms of how this suppression is mediated have been proposed, further experimentation will be necessary to fully elucidate the impact of VISTA on T cells in the TME.

### Suppression of T-cell activation and inhibition of antitumor immunity

Within the T lymphocyte compartment, VISTA is expressed most prominently by naïve CD4^+^ T cells and FoxP3^+^ regulatory T (Treg) cells, with lower expression by CD4^+^ memory and CD8^+^ T cells^2^. In naïve CD4^+^ T cells, VISTA has been shown to act as a negative checkpoint receptor to maintain cell quiescence and peripheral tolerance^9^. Stimulation of VISTA signaling in naïve CD4^+^ T cells with an agonistic antibody augments T-cell tolerance to antigen-specific T-cell receptor signaling^9^. Conversely, the genetic deletion of VISTA in naïve CD4^+^ T cells results in enhanced memory-like phenotypes, enhanced T-cell receptor signaling, and reduced expression of naïve and quiescent phenotypic markers compared with those in wild-type cells. Accordingly, aged VISTA-deficient mice present a greater frequency of peripheral activated T cells and increased susceptibility to autoimmune disease^10,11^. In addition to acting as a receptor, VISTA also acts as a ligand through a putative receptor on the surface of CD4^+^ and CD8^+^ T cells^2^. The engagement of this putative receptor by a VISTA-immunoglobulin fusion protein (VISTA-Ig), VISTA expressed on APCs, or possibly VISTA expressed on neighboring T cells suppresses CD4^+^ T-cell activation^8^. This suppression is, in part, mediated by an impairment of early T-cell receptor signaling^12^.

Because T cells are often regarded as one of the main effectors of antitumor immunity, the inhibition of T-cell activation and maintenance of quiescence can have a negative impact on the control of tumor progression. In a murine model of glioma, VISTA-deficient mice presented a significantly greater frequency of activated CD4^+^ T cells in tumors than did wild-type mice, suggesting an increase in tumor-specific T cells^8^. In a melanoma model, treatment of tumor-bearing mice with a VISTA-blocking antibody synergized with a tumor vaccine to more effectively inhibit the growth of established tumors. Analysis of tumor-infiltrating T cells revealed marked increases in IFN-γ production in both the CD4^+^ and CD8^+^ subsets in this treatment group, suggesting that VISTA blockade may prime T cells to be more effectively activated by a tumor vaccine^5^. Furthermore, its ligand function points to the expression of VISTA by cancer cells as a potential mechanism of tumor immune evasion. In one study, when the endogenous expression of VISTA in human endometrial or ovarian cell lines was silenced, cocultured T cells displayed increased inflammatory activation, suggesting that VISTA expressed on the surface of tumor cells could interact with a putative receptor on T cells to suppress T-cell activation^13^.

Although few studies have directly investigated the function of VISTA expression in human cancer, the available evidence suggests that human VISTA does indeed play a role in suppressing human T-cell antitumor activity in the TME. The frequency of VISTA-positive immune cells within human clear cell renal cell carcinoma tumors is negatively associated with the frequency of tumor-infiltrating CD8^+^ T cells and granzyme B/perforin-positive or TNF-α-positive T cells, suggesting that intratumoral VISTA may suppress T-cell function^14^. In a comparison of human pancreatic cancer and melanoma samples, pancreatic cancer samples were found to express VISTA more highly while also displaying significantly lower numbers of T cells than melanoma samples do, suggesting that higher VISTA expression may contribute to decreased T-cell infiltration in pancreatic tumors^15^. Together, these studies suggest that targeting human VISTA in cancer may be a potential method for enhancing T-cell antitumor activity and improving clinical outcomes.

### Maintenance of regulatory T cells and inhibition of antitumor immunity

VISTA plays a role in the de novo generation and maintenance of induced Treg cells (iTregs), possibly through the suppression of proinflammatory cytokines^16^. However, whether this effect is facilitated through the cell-intrinsic receptor functions of VISTA expressed by T lymphocytes or through VISTA functions when expressed by other immune cells in the TME is unclear. For example, the loss of VISTA in iTreg cells directly led to the instability of Foxp3 expression through epigenetic changes, which subsequently led to increased differentiation into inflammatory T cells, suggesting that VISTA can have a cell-intrinsic impact on the Treg phenotype. Other studies have shown that in vitro VISTA-Ig treatment results in increased conversion of naïve CD4^+^ T cells into iTreg cells, suggesting that VISTA can act as a ligand to induce the Treg phenotype through a putative receptor as well^5,6^. The increased generation of iTreg cells represents an important mechanism of tolerance and immune escape in tumors. Thus, increased VISTA expression by Treg cells in certain cancers may reflect the role of VISTA in inducing and maintaining Treg cells in the TME. Indeed, in vivo VISTA blockade in a melanoma model resulted in diminished tumor-mediated induction of iTreg cells, which coincided with slowed tumor growth and an improved antitumor immune response^5^. In contrast, the overexpression of VISTA in melanoma cells coincided with accelerated tumor onset when inoculated into mice, resulting in a more immunosuppressed TME that contained an increased frequency of Treg cells^17^.

There is some evidence supporting this induction of Treg cells as a mechanism of tumor immune escape in human cancer. Immunohistochemical analysis of melanoma samples revealed that VISTA expression within the tumor area was strongly correlated with FOXP3 expression, an indicator of the presence of Treg cells^18^. Paired samples from patients with melanoma collected either before treatment or after disease progression revealed that the densities of VISTA- and FOXP3-expressing cells were both significantly increased in tumors after disease progression. Furthermore, an analysis of RNA sequencing data from patients with pancreatic ductal adenocarcinoma revealed that a subset of tumors presented both elevated VISTA mRNA expression and elevated expression of Treg cell marker expression, suggesting that VISTA expression is correlated with the presence of Treg cells in these tumors as well^19^. Together, these studies identify the emerging role of VISTA as a negative checkpoint regulator of T cells.

## Negative checkpoint regulation of myeloid cells in cancer

Within the TME, myeloid cells make up a major portion of the immune cell population and are key contributors to antitumor immunity. However, cancer cells can exploit myeloid cells to evade immune surveillance by inducing anti-inflammatory phenotypes, which can ultimately facilitate immune tolerance in the TME. In addition to its role in T cells, studies have identified VISTA as a negative checkpoint regulator of innate immunity that functions to suppress myeloid immune responses. Compared with the T lymphocyte compartment, VISTA is more highly expressed by myeloid lineage cells, including monocytes, macrophages, neutrophils, and DCs^6^. Accordingly, in human cancer, VISTA is also expressed most often by these myeloid immune cell populations^15,20–23^. Thus, investigating how VISTA helps shape immune tolerance in these cell populations will be critical for understanding the mechanisms of tumor immune surveillance and escape.

### Suppression of innate immunity and inhibition of antitumor immunity

In the TME, innate immune cells can promote antitumor immunity by activating the adaptive immune response or even by exhibiting direct tumoricidal functions. The release of chemokines and cytokines can aid the infiltration of leukocytes into the TME and enhance the activity of effector cells. Thus, the regulation of innate immunity by VISTA can play a major role in the initiation and maintenance of antitumor immunity.

In multiple subsets of myeloid cells, VISTA has been shown to be a negative regulator of innate inflammation. In macrophages, VISTA has been shown to suppress the immune response to Toll-like receptor (TLR) stimulation by dampening downstream signaling cascades, ultimately resulting in decreased TLR-mediated cytokine release^7,10^. Intriguingly, one study revealed that the cytoplasmic tail of VISTA is not essential for its suppressive effect, suggesting that VISTA may actually act as a ligand that engages with an unknown receptor to deliver this inhibitory signal^10^. However, other studies have also shown that direct activation of VISTA as a receptor on macrophages with an agonistic antibody can confer a tolerogenic and anti-inflammatory transcriptional profile, and these VISTA-reprogrammed macrophages are subsequently more resistant to proinflammatory activation^24^.

These myeloid-suppressive mechanisms likely contribute to the inhibition of antitumor immunity. In a melanoma model, loss or blockade of VISTA in mice resulted in a TLR signaling-dependent delay in tumor growth and increases in cytokine and chemokine expression in the TME^7^. Furthermore, in a murine model of acute myeloid leukemia (AML), conditional deletion of VISTA in myeloid cells resulted in a significant reduction in leukemia progression^25^. In patients with clear cell renal cell carcinoma, Hong et al. reported an increased frequency of VISTA positivity among CD14^+^HLA–DR^+^ monocytes/macrophages in tumor tissue compared with nontumor samples from the same patient. The frequency of VISTA^+^ cells within tumors was negatively associated with both the frequency of tumor-infiltrating CD8^+^ T cells and the function of these T cells, as measured by granzyme B/perforin positivity or TNFα positivity, suggesting that monocyte/macrophage-expressed VISTA may impair the activation of adaptive antitumor immunity^14^. Taken together, these findings suggest that therapeutic VISTA antagonism may activate myeloid immune cells in cancer and indirectly facilitate the adaptive antitumor immune response.

### Reprogramming of myeloid populations and inhibition of antitumor immunity

Classically, myeloid cells can play a largely beneficial role in antitumor immunity through the recognition of cancer cells, the activation of innate immune pathways, and the priming of adaptive immune responses. However, myeloid cells can display a wide array of phenotypes, and the TME is often populated by reprogrammed myeloid cells, which can promote tumor growth and suppress antitumor immune responses. Current evidence suggests that VISTA-mediated mechanisms may contribute to the establishment of these immunosuppressive myeloid populations in the TME. VISTA can contribute to the chemokine responsiveness of myeloid cells and the chemotaxis of myeloid-derived suppressor cells (MDSCs) to tumors, resulting in a greater population of immunosuppressive cells in the TME and impaired antitumor immunity^26^. VISTA may also help determine macrophage fate by directing transcriptional programming and restraining the expression of proinflammatory (M1-like) phenotype effector genes, thus tipping the balance toward anti-inflammatory (M2-like) phenotypes during macrophage activation and polarization^24^.

A study of human colorectal carcinoma demonstrated VISTA expression by MDSCs. The authors reported that VISTA was expressed in all tested subsets of myeloid cells, including MDSCs, and VISTA expression in intratumoral MDSCs was increased compared with that in MDSCs isolated from matched peripheral blood mononuclear cells^27^. A separate immunohistochemical analysis of oral squamous cell carcinoma samples revealed that VISTA was more highly expressed by tumor-infiltrating immune cells than by immune cells in normal or dysplastic mucosal tissues. Furthermore, the expression of VISTA in tumor tissues correlated with the expression of MDSC markers (CD11b and CD33), suggesting a link between VISTA expression and the presence of intratumoral MDSCs^28^.

Indeed, VISTA has been shown to be crucial for the suppressive function of monocytic MDSCs and tumor-associated subsets of dysfunctional DCs, as VISTA blockade both augmented their secretion of proinflammatory IL-12 and impaired their ability to suppress T-cell activation^7^. In patients with AML, VISTA was found to be overexpressed by circulating MDSCs. An ex vivo coculture of CD8^+^ T cells with MDSCs isolated from patients with AML demonstrated that VISTA knockdown in these MDSCs resulted in significantly decreased T-cell suppression^23^. VISTA expressed on the surface of myeloid cells may also act as a ligand to directly suppress T-cell immune responses through a putative receptor on T cells, as both VISTA-Ig- and VISTA-overexpressing DCs can suppress T-cell activation^2^. However, the loss of MDSC-suppressive function upon VISTA antibody blockade was shown to be dependent on alterations in myeloid cell-intrinsic signaling pathways, suggesting that VISTA regulates myeloid-intrinsic functions to indirectly induce T-cell suppression^7^. Further studies will be necessary to conclusively differentiate how each of these mechanisms may contribute to the suppressive function of tolerogenic myeloid populations in the TME.

Hypoxia within the TME can blunt antitumor immunity. Notably, hypoxia has been shown to induce VISTA expression in myeloid cells and MDSCs through direct upregulation of VISTA transcription via the transcription factor HIF-1α, a key mediator of the cellular response to hypoxia^29^. Deng et al. reported that this hypoxia-induced expression of VISTA in MDSCs contributed to the suppression of T-cell activity. These findings support the crucial role of MDSC-suppressive functions in the TME and suggest an additional mechanism of tumor immune evasion.

VISTA expressed by cancer cells themselves has also been implicated in the promotion of tolerogenic myeloid cells. Using an in vivo model of melanoma, one study demonstrated that VISTA overexpression in tumor cells resulted in increased expression of PD-L1 by tumor-associated macrophages and MDSCs isolated from tumor tissues^17^. Another study reported that in an ovarian cancer model, mice bearing VISTA-overexpressing tumors presented increased accumulation of MDSCs in both tumor tissue and the spleen, which coincided with decreases in the number of tumor-infiltrating CD8^+^IFN-γ^+^ T cells and in animal survival^13^. Continued investigations will be necessary to fully elucidate the mechanisms responsible for these observations.

## Targeting VISTA to overcome resistance to current immune checkpoint inhibitors in cancer

Immune checkpoint inhibitors such as anti-PD-1/PD-L1 and anti-CTLA-4 antagonists, which function by activating the adaptive immune system against tumor cells, have revolutionized cancer therapy. However, significant subsets of patients are nonresponsive to these immunotherapies or develop relapsed disease over time. Although the mechanisms responsible for resistance to current immune checkpoint inhibitors are still incompletely understood, strategies actively being explored to overcome this resistance include the targeting of other novel immune checkpoint molecules or the use of combinatorial therapies. VISTA has been proposed as a target of interest for these purposes, and the use of VISTA blockade in combination therapies is supported by studies reporting the upregulation of VISTA in patients after treatment with immunotherapies, such as anti-PD-1/PD-L1 or anti-CTLA-4, suggesting that VISTA may be associated with resistance to these immune checkpoint inhibitors^18,20^.

Early evidence revealed that VISTA and PD-1 regulate T-cell responses both nonredundantly and synergistically^12^. Furthermore, combinatorial blockade of VISTA and PD-1 in tumor-bearing mice resulted in significantly enhanced tumor clearance compared with single-agent VISTA or PD-1 blockade alone^12^. Combined knockdown of VISTA and CTLA-4 in tumor cells led to increased expression of proinflammatory cytokines in cocultured T cells in vitro, suggesting that the expression of either VISTA or CTLA-4 by tumor cells may significantly inhibit T-cell activity^30^. In a study that utilized mice bearing CT26 colon carcinoma tumors with complete resistance to PD-1/CTLA-4 blockade, the addition of VISTA blockade led to the rejection of more than half of the tumors^31^. This study also revealed that the ability of VISTA blockade to overcome resistance to anti-PD-1/CTLA-4 therapy was due to reductions in both myeloid-mediated suppression and T-cell quiescence in the TME. In a murine AML model, the use of combined VISTA and PD-1 blockade resulted in a synergistic antileukemic effect significantly greater than either PD-1 or VISTA blockade alone and nearly complete clearance of leukemia^25^. Together, these studies provide a rationale for the inclusion of VISTA blockade in combinatorial immunotherapies to help overcome resistance to current immune checkpoint inhibitors in cancer.

## Other VISTA functions relevant to cancer

Although VISTA has predominantly been described as an immunosuppressive cosignaling molecule, studies have also shown other possible roles for VISTA. Further investigations into these functions may reveal additional insights into how VISTA may impact the TME.

### Engulfment of apoptotic cells

VISTA has been shown to be involved in the engulfment of apoptotic cells through homophilic interactions between VISTA on the surfaces of apoptotic cells and phagocytes. P53 activity induced the expression of VISTA in apoptotic cells to mediate the efficient clearance of dead cells by phagocytes; however, the loss of VISTA in either apoptotic cells or phagocytes resulted in diminished engulfment. Notably, phagocyte-expressed VISTA was shown to only mediate the engulfment of VISTA-expressing targets, and the loss of VISTA in macrophages did not affect their ability to phagocytose either synthetic beads or bacteria^32^. This ability to facilitate apoptotic cell clearance was also observed in HIV-infected T cells. Both VISTA upregulation in T cells during apoptosis and VISTA expression by phagocytes contribute to the engulfment of apoptotic, HIV-infected T cells^33^. This process of apoptotic cell engulfment, also known as efferocytosis, is generally regarded as an anti-inflammatory process whereby the clearance of cell corpses and downstream induction of immune tolerance prevent the accumulation of autoantigens and ensure tissue homeostasis. In contrast, studies have shown that phagocyte engulfment by target cells can also be proinflammatory. Phagocytes may alternatively engulf and kill live target cells while triggering proinflammatory effectors via the cross-presentation of target cell antigens^34^. Although current evidence suggests that VISTA-mediated efferocytosis is an anti-inflammatory process, much like the other described immunosuppressive functions of VISTA, these studies are primarily in the context of autoimmune disease. Further studies are needed to clarify whether this process also contributes to immune tolerance in the TME or if it may alternatively serve as a proinflammatory process that can activate adaptive antitumor immunity.

### Cellular differentiation

A few early studies investigated the role of VISTA as a regulator of cell differentiation in mouse embryonic stem cells and preadipocytes and demonstrated that VISTA expression is required for these cells to differentiate into more mature cell types^35,36^. The cellular differentiation state and stemness of tumor cells play significant but complex roles in both tumorigenesis and cancer progression, and investigating how VISTA may govern cell differentiation could provide insights into the role of VISTA in cancer. An in vitro model of epithelial‒mesenchymal transition (EMT), a developmental program that may provide cancer cells with stem cell-like properties and promote resistance to treatment, demonstrated that dedifferentiation into mesenchymal cells was associated with a loss of VISTA expression^37^. VISTA expression was also found to be inversely correlated with the expression of EMT-associated genes in malignant pleural mesothelioma tumors, thus supporting the notion that VISTA expression is downregulated during EMT^38^. Furthermore, the stemness factor forkhead box D3, which has previously been shown to mediate resistance to melanoma therapy, was found to directly repress VISTA transcript and protein expression in melanoma cells^17^. Together, these studies illustrate a possible connection between VISTA and the cellular differentiation state, where VISTA may promote cell differentiation and maturation. Further elucidation of the mechanisms of VISTA in these developmental processes may provide a clearer understanding of the overall impact of VISTA on cancer progression, including possible tumor cell-intrinsic roles that extend beyond its proposed functions as a checkpoint ligand.

### Osteoclast activation

Matrix metalloproteinase 13 (MMP-13) was previously established as a key factor in triggering osteoclast-mediated bone resorption^39^. A recently published study identified a novel role for VISTA as a receptor for MMP-13 on osteoclast precursors, whereby the binding of VISTA induced osteoclast formation and activation, leading to bone resorption^40^. In the context of multiple myeloma, in which MMP-13 can be highly expressed by malignant cells, this MMP-13/VISTA signaling axis can mediate excessive osteoclast activation and the development of osteolytic bone disease. Moreover, osteoclasts are not only derived from monocyte/macrophage lineage cells but also able to function as APCs to either activate CD4^+^ and CD8^+^ T cells or even promote immune suppression^41,42^. Whether VISTA expression on osteoclasts contributes to an immunosuppressive role remains to be determined.

## VISTA as a prognostic factor in cancer

The interpretation of observational studies in human cancer can be difficult, particularly with respect to the relationship between VISTA expression and patient prognosis. For example, because VISTA is expressed primarily by immune cells, the high VISTA expression observed in a tumor could represent a more immunologically active TME with increased immune cell infiltration rather than being a marker for VISTA-mediated immune suppression. Accurate prognostic associations would require extensive analyses of TME factors paired with functional studies to confirm the mechanisms responsible for any observations. Thus, although numerous groups have attempted to define these associations between VISTA expression in cancer and clinical prognosis, the findings vary greatly, and VISTA expression has been proposed to be either a negative or positive predictor of prognosis (Table 1). Few studies have provided strong and convincing evidence in either direction. Ultimately, a clear understanding of the mechanisms of VISTA in cancer will likely be necessary before the impact of VISTA expression on clinical outcomes can be conclusively defined.

**Table 1:** List of studies that have assessed VISTA expression in human cancer.

Cancer type	Analysis type (sample size)	Prognosis associated with VISTA expression (cell type assessed)	Change in VISTA expression; vs. nontumor control if not specified	Cell type expression (frequency of samples)	Functional studies	Ref.
AML	CyTOF of bone marrow (14)			ICs, leukemic blasts		^50^
AML	IHC (10) and FC (6) of bone marrow		Up in leukemic blasts	ICs, leukemic blasts	In vivo AML model	^51^
AML	FC of blood (30)	n.s.	Up in MDSCs	ICs, leukemic blasts	Ex vivo MDSC functional assay	^23^
AML	Publicly available RNAseq (672) and scRNAseq (9) datasets	Worse (unspecified)	Up	ICs, leukemic blasts		^52^
AML	TCGA dataset (162), IHC (21), and FC (26) of bone marrow	Worse (TCs) by TCGA dataset	Up in leukemic blasts	ICs primarily myeloid, leukemic blasts (90% by IHC)	In vivo AML model, humanized AML mouse model	^25^
Bladder	IHC of TMA (135), TCGA dataset (391), and publicly available dataset (195)	Worse (ICs)		ICs, TCs (27%)		^53^
Breast	IHC of TMA (919)	Better (ICs)		ICs (29%), TCs (8%)		^54^
Breast	IHC of TMA (254)	Better (ICs)		ICs (88%), TCs (19%)		^55^
Clear cell renal cell	IHC and FC of tissue (47)		Up	ICs, TCs (lower)	In vivo tumor model	^14^
Colorectal	IHC (32) and FC (14) of tissue		Up in gMDSCs	ICs		^27^
Esophageal	IHC of TMA (393)	Better (unspecified)		ICs (22%), TCs (1.2%)		^22^
Gastric	qRT-PCR of tissue (45)		Down	Unspecified		^37^
Gastric	IHC of tissue (464)	n.s.		ICs (84%), TCs (9%)		^56^
Glioma	qRT-PCR (87) and IHC (30) of tissue	Worse (unspecified)	Up	ICs (20%), TCs (most)		^57^
Hepatocellular	IHC of TMA (183)	Better (TCs)		ICs (17%) or TCs (16%)		^58^
Lung	qIF of TMA (636)	Better (unspecified)		ICs (most), TCs (21%)		^59^
Lung	IHC of tissue (66)	n.s.		ICs, lymphocytes (20%)		^60^
Melanoma	IHC of tissue (18)		Up after disease progression	ICs		^18^
Melanoma	IHC of tissue (85)	Better (unspecified)		ICs (62%)		^21^
Melanoma	Publicly available dataset (186) IHC of TMA (676) FC of tissue (20)	Worse (CTL low subset)		ICs, TCs (lower)	Multiple in vitro and in vivo studies	^17^
Melanoma, uveal	TCGA dataset (80) and IHC of tissue (25)	Worse (TCGA data)		ICs (76%), TCs (72%), ECs (16%)		^61^
Melanoma	qIF of TMA (190)	Worse (CD11b^+^ ICs)		ICs (55%) primarily myeloid, TCs (36%)		^62^
Mesothelioma	Multiplatform analysis of tissue (74)			ICs, TCs		^38^
Mesothelioma	IHC of TMA (161)	Better (TCs)		ICs, TCs		^63^
Mesothelioma	IHC of TMA (319)	Better (TCs)		ICs, TCs		^64^
Oral squamous cell	IHC of TMA (165)	Worse (CD8 low subset)	Up	ICs		^28^
Oral squamous cell	IHC of TMA (75)	Worse (MDSCs)	Up	ICs	In vivo tumor models	^65^
Ovarian, endometrial	IHC of ovarian (92) or endometrial (82) tissue	n.s.	Up	ICs, TCs	In vitro and in vivo tumor models	^13^
Ovarian	IHC of TMA (146)	Better (TCs)		ICs or TCs (some)		^66^
Pancreatic	Publicly available dataset (134)			Unspecified		^19^
Pancreatic	IHC of tissue (52)		Up	ICs (88%), TCs (8%)		^67^
Pancreatic, melanoma	IHC of pancreatic (23) or melanoma (44) tissue	Worse (unspecified)	Up in pancreatic vs. melanoma	ICs, primarily macrophages	Ex vivo T-cell suppression assay	^15^
Pancreatic	IHC (76) and IF (67) of tissue, FC of matched tumor and blood samples (13)	n.s. (TCs), trend toward worse	Up in TCs	ICs, TCs	In vitro T-cell suppression, in vivo tumor model	^68^
Pancreatic	Publicly available datasets (253) and IHC of TMA (228)	n.s.	n.s.	ICs or TCs (76%), TCs (16%)		^69^
Pancreatic, intraductal papillary mucinous neoplasm	IHC of tissue (60)	n.s. (ICs)		ICs, TCs (11–12%)		^70^
Prostate	IHC of tissue (10)		Up after ipilimumab treatment	ICs	Ex vivo T-cell suppression assay	^20^
Synovial sarcoma	IMC (9) and IF of tissue (66)	Worse in metastatic disease (ECs)	Up in ECs	ICs, TCs (0–3%), ECs (71–100%)	In vitro T-cell migration	^71^

Blank cells indicate that a parameter was not assessed or reported in the study.

*AML* acute myeloid leukemia, *CyTOF* mass cytometry, *IHC* immunohistochemistry, *FC* flow cytometry, *RNAseq* RNA sequencing, *scRNAseq* single-cell RNA sequencing, *TMA* tissue microarray, *qRT-PCR* quantitative reverse transcription polymerase chain reaction, *IF* immunofluorescence, *qIF* quantitative immunofluorescence, *IMC* imaging mass cytometry, *n.s.* no significant difference, *ICs* immune cells, *TCs* tumor cells, *ECs* endothelial cells, *CTLs* cytotoxic T lymphocytes, *MDSCs* myeloid-derived suppressor cells, *gMDSCs* granulocytic MDSCs.

## VISTA binding partners

The search for relevant binding partners to VISTA is ongoing. Gene editing and antagonistic or agonistic antibodies have enabled the interrogation of VISTA signaling mechanisms, though there has been limited success in the discovery of receptor- or ligand-binding partners. Early studies proposed that VISTA associated with Alk3 on the surface of mouse embryonic stem cells to act as a coreceptor for bone morphogenetic protein 4^43^. Others have described homophilic interactions between VISTA expressed by apoptotic cells and macrophages as well as a role for osteoclast-expressed VISTA as a receptor for MMP-13 in the context of myeloma-associated osteolysis^32,40^. While intriguing, these proposed binding partners largely do not address the prominent immune suppressive functions of VISTA.

A landmark study revealed that VISTA is an acidic pH-selective ligand of P-selectin glycoprotein ligand-1 (PSGL-1), which is expressed on the surface of T cells and can suppress T-cell function^44^. This pH-selective interaction is especially relevant in the context of the TME, which is often acidic, and even suggests a novel strategy to target tumor acidity. Although previous studies have shown that PSGL-1 is an inhibitory immune checkpoint upregulated by activated CD8^+^ T cells, PSGL-1 is also known to be expressed at steady state by most lymphoid and myeloid cells, including hematopoietic stem and progenitor cells^45,46^. Therefore, future studies investigating the role of the PSGL-1/VISTA interaction in other immune subsets may help to clarify the immunosuppressive mechanisms of VISTA. V set and immunoglobulin containing 3 (VSIG-3) was also identified as a ligand of VISTA, and engagement of the VISTA receptor on T cells by VSIG-3 inhibited T-cell proliferation and inflammatory activation^47^. However, normal expression of VSIG-3 is restricted to brain and testis tissues, and though VSIG-3 upregulation has been observed in gastric and hepatocellular carcinomas, its expression has not been reported in normal immune cells, limiting the relevance of this VISTA binding partner in other settings or cancers. Another study demonstrated a specific interaction between VISTA and galectin-9, a known ligand that can be secreted by AML cells, with the negative checkpoint receptor TIM-3. VISTA can act as a receptor for galectin-9 to inhibit granzyme B release and induce apoptotic cell death in Jurkat T cells. However, further studies are still needed to validate this mechanism in primary immune cells and determine whether this VISTA/galectin-9 interaction also impacts other T-cell functions in addition to granzyme B release^48^. Although each of these studies proposed how ligand‒receptor interactions with VISTA could impact immune evasion in cancer, clear roles for these interactions in broader or nonpathologic settings have not yet been elucidated. Thus, how these or other undiscovered binding partners may contribute to the homeostatic and tolerance-inducing functions of VISTA is still poorly understood and requires further investigation.

## Conclusion

**Fig. 1:**
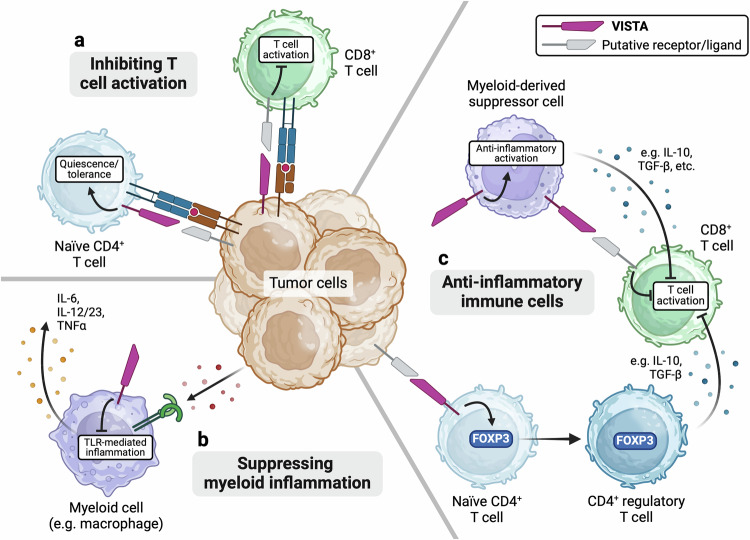
Immune mechanisms of VISTA in cancer. **a** VISTA can directly inhibit the antitumor response in T cells. In naïve CD4^+^ T cells, VISTA can act as a receptor that maintains a quiescent or tolerant phenotype. Alternatively, VISTA may act as a ligand expressed by tumor cells that signal through a putative ligand on T cells to inhibit T-cell receptor-mediated activation. **b** VISTA can inhibit inflammatory activation in myeloid immune cells. Myeloid cells can recognize and respond to damage-associated molecular patterns released by cancer cells through Toll-like receptors (TLRs), which in turn recruits and activates the adaptive immune response. VISTA may inhibit downstream signaling from TLRs, thus suppressing the TLR-mediated production of proinflammatory cytokines and preventing the activation of adaptive antitumor immunity. **c** VISTA can augment immunosuppressive cell populations in the TME, which in turn impairs antitumor immunity. The expression of VISTA by myeloid-derived suppressor cells enhances their ability to suppress T-cell activity. Furthermore, VISTA signaling in naïve CD4^+^ T cells may stabilize FOXP3 expression and contribute to the maintenance of regulatory T cells in the TME. Created with BioRender.com.

**Fig. 2:**
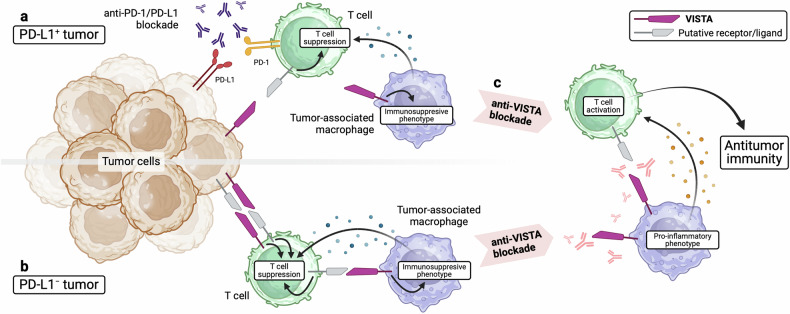
Anti-VISTA blockade overcomes resistance to anti-PD-1/PD-L1 (anti-PD) therapy. **a** In PD-L1^+^ tumors, VISTA may contribute to primary or acquired resistance to anti-PD therapy through the augmentation of immunosuppressive cell populations, including immunosuppressive myeloid immune cells. Tumor cells may also express or upregulate VISTA to inhibit T-cell antitumor activity through a putative receptor. **b** In PD-L1^−^ tumors, anti-PD therapy is ineffective due to the lack of a target. T-cell suppression may instead be mediated by alternative immune checkpoint pathways, including the use of VISTA as either a receptor or a ligand. VISTA may augment immunosuppressive cell populations in the TME or may directly suppress T-cell activity. **c** Anti-VISTA blockade, especially as part of combination therapy, alters the phenotypes of myeloid immune cells within the TME such that they are more proinflammatory and may enhance the activation of T cells, ultimately resulting in an improved antitumor immune response. Created with BioRender.com.

VISTA is an immune-regulatory molecule with broad expression and multifaceted roles within the immune system. Describing it solely as an inhibitory immune checkpoint would diminish its diverse functions within the body and could hinder our understanding of its role in cancer. VISTA has been identified as a critical mediator of cancer immune tolerance through its mechanisms of inhibiting the activation of tumor-responsive T cells and myeloid cells in addition to enhancing the presence and function of tolerogenic immune populations in the TME (Fig. 1). Moreover, the potential regulatory roles of VISTA in phagocytosis, cellular differentiation, and osteoclast activity may have additional implications for how VISTA impacts tumor progression and immunity. Importantly, VISTA expression has been shown to be relevant in numerous types of human cancer, both expressed by immune cells and occasionally by cancer cells themselves. Thus, the ability of VISTA to regulate both cell-intrinsic processes and the TME, together with its broad and unique expression pattern, identifies it as a key regulator of tumor immune surveillance. Furthermore, the parallels between the immunosuppressive roles of VISTA in innate and adaptive antitumor immunity make VISTA a promising target for the development of novel immunotherapies (Fig. 2). The ability to modulate the functions of multiple immune cell populations could elicit synergistic effects and warrants further investigation into the utility of VISTA antagonism in cancer. Although clinical trials have already been initiated to evaluate VISTA as a target of cancer immunotherapy (reviewed by Martin et al.^49^), further research will be necessary to fully understand the role of VISTA in cancer and guide the development of more effective therapies.
